# Large Solitary Lung Metastasis of a Matrix-Producing Metaplastic Breast Carcinoma: A Diagnostic Challenge

**DOI:** 10.7759/cureus.50265

**Published:** 2023-12-10

**Authors:** Rida Noor, Sigfred Lajara, Rohit Bhargava

**Affiliations:** 1 Department of Pathology, Faisalabad Medical University, Faisalabad, PAK; 2 Department of Pathology, UPMC (University of Pittsburgh Medical Center) Presbyterian-Shadyside Hospital, Pittsburgh, USA; 3 Department of Pathology, UPMC (University of Pittsburgh Medical Center) Magee-Womens Hospital, Pittsburgh, USA

**Keywords:** metastasis, invasive breast carcinoma, matrix-producing carcinoma, myoepithelial cell, metaplastic breast cancer

## Abstract

Metaplastic breast carcinoma represents a diverse category of invasive breast cancers distinguished by the transformation of neoplastic epithelial cells into squamous cells or cells with mesenchymal appearance. Matrix-producing breast carcinoma is a variant of metaplastic breast carcinoma, an exceedingly uncommon malignancy accounting for less than 1% of all breast tumors. The precise origin of this tumor remains elusive; some molecular research points to a potential derivation from myoepithelial cells, while other studies emphasize the possibility of neoplastic transformation originating from multipotent stem cells.

We report a case of recurrent matrix-producing breast carcinoma. The patient presented with a breast mass. The tumor cells displayed a lack of reactivity for estrogen receptor (ER), progesterone receptor (PR), and human epidermal growth factor receptor-2 (HER2), and exhibited a Ki-67 proliferation index of approximately 40%. Additionally, the tumor cells demonstrated significant reactivity for cytokeratins and S100. The patient underwent surgery, radiation, and chemotherapy and then developed metastasis to the lower lobe of her left lung, seven years after primary diagnosis. Diagnosis of metastasis was confirmed by comparing the metastasis to the primary tumor and staining with a panel of immunohistochemical stains. The patient is currently undergoing chemotherapy and immunotherapy.

## Introduction

Metaplastic breast carcinomas are an infrequent and diverse category of diseases, accounting for approximately 0.3% to 5% of all breast cancer cases [1]. There are several morphological subtypes of metaplastic breast carcinomas, including squamous cell carcinoma, spindle cell carcinoma, and metaplastic carcinoma with heterologous mesenchymal differentiation (also known as matrix-producing metaplastic carcinoma) [2]. Metaplastic breast carcinoma can be categorized into various subgroups based on its histological features, biological characteristics, and prognosis. Within the metaplastic carcinoma subgroup, a distinct type is characterized by the presence of both ductal and mesenchymal components, including elements like bone, cartilage, and fibrous tissue or striatum, embedded in a rich extracellular matrix. Lymph node involvement is less frequent compared to non-metaplastic histotypes and the hormone receptor expression is often negative. Metastasis of matrix-producing cancer (MPC) through the lymphatic system is a rare occurrence, as it typically tends to prefer blood-borne metastasis [3].

## Case presentation

**Figure 1:**
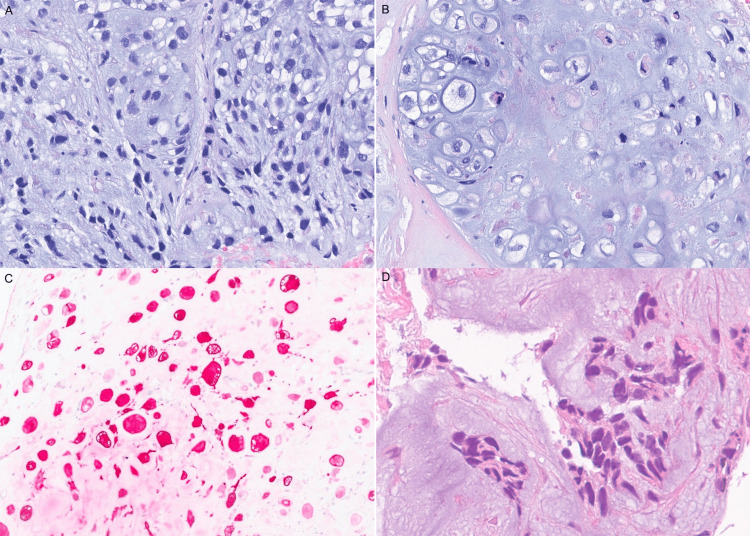
The primary breast carcinoma showed malignant hyperchromatic cells with extracellular basophilic matrix (A, H&E stain), with some areas resembling chondrosarcoma (B, H&E stain). The tumor cells were positive for S100, supporting the histomorphologic impression of cartilaginous differentiation (C, S100 stain, fast red used as chromogen). The metastatic tumor in the lung showed features similar to the breast primary tumor (D, H&E stain). H&E: hematoxylin and eosin.

**Figure 2:**
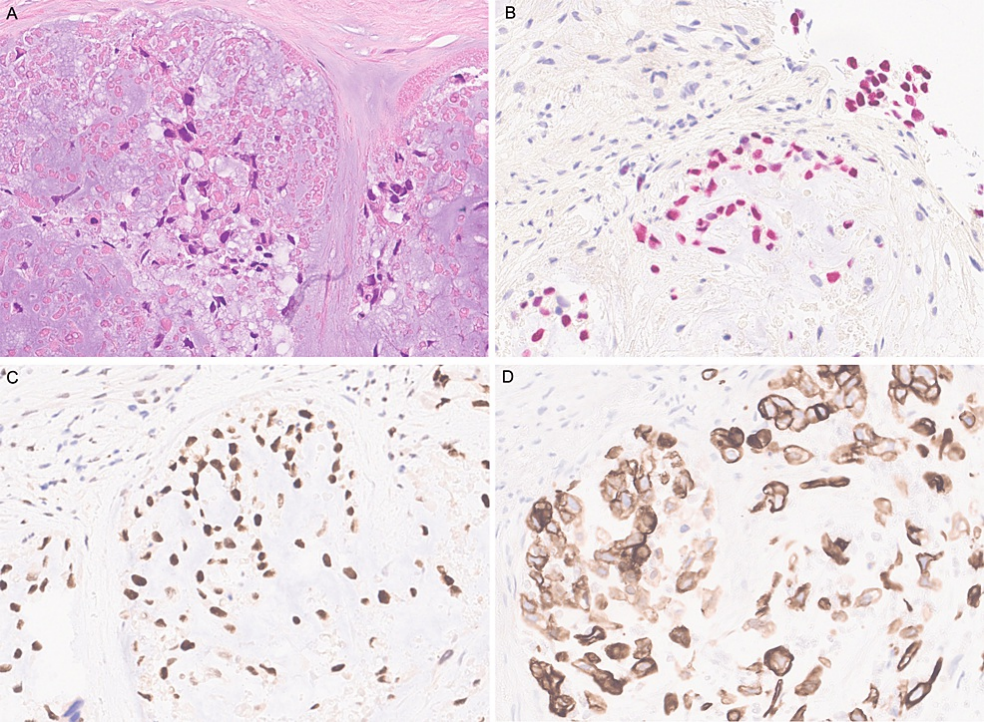
The metastatic tumor in the lung showed scattered hyperchromatic cells with abundant necrosis and basophilic matrix (A, H&E stain). The tumor cells showed reactivity for SOX10 (B, SOX10 stain, fast red used as chromogen), sensitive breast marker TRPS1 (C, TRPS1 stain, DAB used as chromogen), and cytokeratin (D, CK8/18 stain, DAB used as chromogen), confirming breast as the site of the origin of lung metastatic cancer. H&E: hematoxylin and eosin; DAB: 3,3'-diaminobenzidine.

A 64-year-old woman presented with a very large unilateral right breast mass with overlying skin ulceration that increased in size over the last several months. The mass was associated with episodic bloody nipple discharge. The patient never had breast surgery in the past. There was no family history of breast and ovarian carcinoma. She was not on hormone replacement therapy. A core biopsy of the mass was performed, which showed a tumor composed of spindle to oval large hyperchromatic cells with scattered foci of necrosis surrounded by amphophilic to basophilic matrix, consistent with a matrix-producing metaplastic carcinoma of the breast (Figure 1). The tumor cells were negative for estrogen receptor (ER), progesterone receptor (PR), and human epidermal growth factor receptor-2 (HER2), and showed a Ki-67 proliferation index of approximately 40%. The tumor cells showed substantial reactivity for cytokeratins (AE1/AE3, CK8/18, CK7) and S100, and only focal reactivity for CK5 and p63. The tumor showed strong reactivity for SOX10 and trichorhinophalangeal syndrome type-1 TRPS1 stains, but weak reactivity for GATA3 and no reactivity for GCDFP-15 and mammaglobin. This immunoprofile was consistent with a matrix-producing metaplastic carcinoma. Positron emission tomography (PET) and computed tomography (CT) scans were negative for any metastatic disease. Since metaplastic carcinomas typically show poor response to neoadjuvant chemotherapy, a decision was made to proceed with primary surgery. Although the patient was clinically node-negative, a modified radical mastectomy was performed. The mastectomy specimen showed a 14 cm matrix-producing metaplastic carcinoma with skin ulceration and 15 negative axillary lymph nodes (tumor stage pT4b pN0). Thereafter, the patient completed adjuvant chemotherapy (adriamycin, cyclophosphamide followed by taxane regimen) and adjuvant post-mastectomy radiation. She remained disease-free for seven years at which time she developed shortness of breath and was discovered to have a 6.2 cm mass in the lower lobe of her left lung. A CT-guided biopsy of the lung mass showed a malignant neoplasm with a chondroid matrix, identical to the patient’s prior metaplastic carcinoma. The lung lesion showed the same immunoprofile as the prior breast carcinoma (Figure 2). These findings were consistent with metastasis of the patient’s known matrix-producing metaplastic carcinoma. The patient had a large metastatic tumor, the removal of which would require a complete lobectomy with significant morbidity. She is currently receiving chemotherapy (taxane) and immunotherapy (pembrolizumab).

## Discussion

Matrix-producing carcinoma of the breast represents a rare subtype of metaplastic breast carcinoma, but hundreds of cases have been documented in the literature [3-10]. The cellular source of matrix-producing carcinoma still lacks clarity, although ultrastructural examination suggests that the tumor cells may arise from both epithelial and myoepithelial cells. Myoepithelial cells are known to undergo differentiation in the direction of mesenchymal characteristics, leading to the production of various matricial patterns [9]. Matrix-producing carcinoma typically manifests around the late 50s to early 60s with a swiftly growing, painless, clearly defined lump [5].

There are limited data on the prognosis of matrix-producing carcinoma. Metaplastic breast cancers in general exhibit a behavior similar to or more aggressive than carcinoma of no special type when patient age, stage, and tumor grade are taken into account [11]. Among metaplastic breast cancers, high-grade spindle and squamous carcinomas tend to be more aggressive than matrix-producing carcinomas [12,13]. In mixed metaplastic carcinomas, the higher number of morphologies in a given tumor may portend a worse prognosis [1,14]. Key factors influencing the prognosis in metaplastic breast carcinoma, including matrix-producing carcinoma, include tumor size and lymph node status. Multivariate analyses have shown worse recurrence-free survival and breast cancer-specific survival associated with higher pathologic tumor (pT) and pathologic nodal (pN) stages [1].

Molecularly, little is known about the genomic landscape of matrix-producing carcinomas. These tend to be either basal-like or mesenchymal subtypes under the Lehmann classification of triple-negative breast cancers [15,16]. In a targeted sequencing study of 28 metaplastic carcinomas (10 matrix-producing and 18 non-matrix-producing), distinct differences were identified between subtypes. Non-matrix-producing metaplastic carcinomas were highly enriched for PIK3CA/PIK3R1 and Ras-Map kinase pathway aberrations, but none of the matrix-producing carcinomas showed such alterations [17]. A recent fluorescence in-situ hybridization (FISH) study of 12 matrix-producing metaplastic breast carcinomas showed PLAG1 and MYC gene rearrangements/amplifications. These alterations are also seen in salivary tumors, especially carcinomas like ex-pleomorphic adenoma (which may also show a cartilaginous matrix production) [18].

Diagnosis of matrix-producing breast carcinoma can be challenging at both the primary as well as the metastatic site. At the primary site, the differential diagnosis includes primary breast sarcomas and adenomyoepithelial neoplasms. Although adenomyoepithelial neoplasm of the breast can produce a matrix, they often show a characteristically bland two-cell population. In contrast, it can be impossible to distinguish matrix-producing carcinomas from primary sarcomas when the epithelial component is focal or if reactivity for epithelial markers is focal or weak. To resolve this dilemma, Rakha et al. conducted an international multi-institutional study where they compared 101 matrix-producing breast tumors to 253 other known subtypes of metaplastic breast carcinomas and 258 hormone-receptor-matched carcinomas of no special type [8]. They found that the majority of matrix-producing carcinomas showed epithelial features, 21% showed nodal metastases, and the distribution of distant metastases resembled conventional mammary carcinoma. The prognosis of matrix-producing tumors was comparable to matched breast carcinomas of no special type and slightly better than other subtypes of metaplastic breast cancer. These findings provided evidence that the majority (if not all) of matrix-producing tumors of the breast represent matrix-producing breast carcinoma and are likely variants of metaplastic breast cancers. Immunohistochemical stains for multiple cytokeratins and epithelial markers can also help confirm the epithelial origin. One should use different types of cytokeratin (particularly high-molecular-weight keratins such as CK5 and CK14) and epithelial markers to confirm epithelial origin. p63 has been identified as a valuable diagnostic indicator for metaplastic cancer. The sensitivity and specificity of p63 in detecting metaplastic carcinoma are reported to be 87% and 99%, respectively [19]. However, it is to be noted that p63 is often weakly expressed or is negative in a matrix-producing subtype of metaplastic carcinoma. Breast-specific markers can also be used to confirm the diagnosis. GATA3 often shows some degree of nuclear reactivity in matrix-producing metaplastic breast carcinomas. TRPS1, a recently described sensitive marker of breast cancer, is also often positive, but caution is advised. Our recent findings suggest that TRPS1 may not serve as a completely specific marker for breast neoplasia [20]. Additionally, markers of basal phenotypes, such as SOX10, may also be useful in confirming the diagnosis. However, staining for SOX10 should always be coupled with cytokeratin staining. Matrix-producing carcinomas are often positive for S100. Since both S100 and SOX10 are also melanocytic markers, we strongly recommend using other melanoma markers (HMB45, Melan A) and cytokeratins to distinguish between melanoma and matrix-producing carcinomas. Just like the primary site, the diagnosis at the metastatic site is similarly challenging. Without prior history, it can be even more difficult to diagnose matrix-producing metaplastic breast cancer. If the patient has a prior diagnosis, it is best to retrieve the prior specimen and compare, which not only helps to confirm the diagnosis of metastasis but also saves effort and resources involved in a comprehensive workup. In the absence of prior breast cancer diagnosis, the differential diagnosis is broad and may include myoepithelial carcinoma and extraskeletal myxoid chondrosarcoma. The latter diagnosis can be excluded by doing EWS gene rearrangement studies by FISH assay. Ultimately, a large panel of immunohistochemical stains (including keratins, epithelial markers, breast-specific markers, and basal markers) may be required for a definitive diagnosis. Ultimately, one should have a high index of suspicion for metastasis given the patient’s prior history.

## Conclusions

Matrix-producing metaplastic breast carcinoma is a rare special subtype with distinct clinical features and molecular profile. Currently, it is treated with multimodality therapy. Specific targetable molecular alterations have not been identified. The prognosis strongly depends on the tumor stage. This carcinoma can metastasize years after initial diagnosis, so long-term surveillance is needed. Diagnosis at primary as well as metastatic sites requires a high degree of suspicion, judicious use of immunohistochemical stains, and familiarity with the diagnostic entity.
